# *Porphyromonas gingivalis*-lipopolysaccharide and amyloid-β: A dangerous liaison for impairing memory?

**DOI:** 10.1177/13872877251343317

**Published:** 2025-05-21

**Authors:** Sim K Singhrao

**Affiliations:** 1 School of Medicine and Dentistry, University of Central Lancashire, Preston, UK

**Keywords:** Alzheimer's disease, amyloid-beta, cognitive decline, inflammation

## Abstract

Alzheimer's disease is characterized by declining memory and the presence of insoluble amyloid-β (Aβ) plaques and neurofibrillary tangles in the brain. Gui et al.^1^ studied the effects of low levels of *Porphyromonas gingivalis*-lipopolysaccharide (*P. gingivalis*-LPS) and soluble Aβ on synaptic proteins, synapsin1 (SYN1) and post-synaptic density protein-95 (PSD-95). Their study revealed increased proinflammatory cytokine production in microglial cells (MG6) treated with *P. gingivalis*-LPS and Aβ, while neuronal cells, N2a, exposed to MG6-conditioned medium showed SYN1 and PSD-95 loss. This suggests that excessive neuroinflammation may contribute to synaptic protein and memory loss, offering mechanistic insights into *P. gingivalis*-LPS-mediated inflammatory pathways in periodontitis.

Gui et al.^1^ highlight the intricate relationship between systemic infection, inflammation, and Alzheimer's disease (AD) by focusing on periodontal disease through the example of *Porphyromonas gingivalis*-lipopolysaccharide (*P. gingivalis*-LPS) and its interaction with amyloid-β (Aβ). Systemic infections are implicated in AD and are strongly linked to worsening cognitive decline. Examples include urinary tract infections, pneumonia, and periodontitis, which are primarily driven by alterations in the host's inflammatory response to pathogens.

Gui et al.^1^ article illustrates that when low levels of *P. gingivalis*-LPS and low levels of Aβ were applied individually over a period of 48–72 h, no detrimental effects were observed on synaptic proteins within neurons at the gene expression level. This suggests that neuronal cells can tolerate low levels of exposure to these noxious agents.

In contrast, the microglial cells in the Gui et al.^1^ article sensitively responded to low levels of exposure to *P. gingivalis*-LPS and Aβ revealing compelling clues to inflammation being initiated. These cells exhibited a spontaneous increase in the production of proinflammatory cytokines, especially tumor necrosis factor-alpha (TNF-α), mediated through the Toll-like receptor (TLR)-2 signaling pathway. As the brains immune guardians, microglia play a crucial protective role against invading pathogens in general, making the cytokine release an anticipated response. While TNF-α contributes to essential processes such as neurogenesis, synaptic plasticity, and the maintenance of the blood-brain barrier,^2^ its excessive or chronic production can result in neuroinflammation, exacerbating the progression of neurodegenerative diseases.^2^

*P. gingivalis*-LPS is an agonist for TLR-2 and can also act as either an antagonist or agonist for TLR-4 activation depending on the specific form of *P. gingivalis*-LPS presented to the host.^3,4^ This variability arises due to the heterogeneity in the lipid A structure of *P. gingivalis*-LPS.^5^ Interestingly, Gui et al.^1^ reported that their application of *P. gingivalis*-LPS exclusively upregulated TLR-2 activation, though they did not implicate TLR-4 activation. However, the implications of TLR-4 activation were shown in the supplementary data of Gui et al.^1^ article, and the *P. gingivalis*-LPS structural implications would have been of interest. The authors specified that the *P. gingivalis*-LPS used in their study was commercially sourced, potentially accounting for the observed selective TLR-2 activation.

Gui et al.^1^ further investigated novel methods of exposing the neuronal cell line N2a to the noxious substances (low levels of *P. gingivalis*-LPS and Aβ) that were first applied to the microglial cell line MG6. Remnants of these noxious substances and additional inflammatory mediators including cytokines released by MG6 cells in response were in the conditioned medium (termed AL-MCM) and were used to treat N2a cells to evaluate synaptic protein loss as an indicator of memory impairment. The anticipated reduction in synapsin1 (SYN1) and post-synaptic density protein-95 (PSD-95) within N2a synapses was confirmed, strongly implicating microglia mediated inflammation in the loss of these proteins.

To clarify the mechanisms, Gui et al.^1^ reported stress related factors such as the production of cellular reactive oxygen species (ROS), were also found to increase in the MG6 cells. While the primary role of cellular ROS is to kill pathogens, they also act as secondary signaling mediators for inflammation and initiating immune responses via the NF-κBp65 signaling pathway.^6^ Nuclear Factor-κB (NF-κB) is further involved in cytokine secretion via the TLRs activation. Gui et al.^1^ demonstrated the activation of this pathway by detecting TLR-2. Additionally, ROS mediated activation of NF-κBp65 was implied, as the authors mentioned p65 protein without explicitly linking it to the NF-κBp65 pathway. Notably, they observed that NF-κB activation was prolonged, creating a ‘double whammy’ effect that led to excessive inflammation, ultimately detrimental to synaptic proteins and memory as demonstrated in their in vivo mouse model investigation.

It is highly likely that the conditioned medium applied to N2a cells to assess synaptic protein loss contained various additional inflammatory mediators such as extracellular adenosine-5’-triphosphate (eATP), a product of ROS mediated cell death. eATP is a recognized ‘danger signal’ molecule,^7,8^ capable of inducing ROS production for pro-inflammatory gene transcription via the transcription factors NF-kB and NF-kBp65 coupling.^9^ Moreover, the Mapk pathways, specifically extracellular signal-regulated kinase (ERK), c-Jun N-terminal kinase (JNK), and p38 pathways are activated by both Aβ and *P. gingivalis*-LPS.^10–12^ The downstream effects of these pathways include the liberation of more cytokines and stress related proteins to combat infection.^13^

Gui et al.,^1^ however, did not address the role of ERK/JNK/p38 stress pathways in their discussion. Additionally, it would have been valuable to explore whether *P. gingivalis*-LPS physically bound to the Aβ in solution, potentially forming insoluble fibrillar structures. This could have provided further insight into their combined toxicity and the resulting inflammatory response. Nevertheless, this study consolidates novel insights that help unravel the distinctive inflammatory pathways mediated by *P. gingivalis*-LPS from the periodontal disease infection perspective.

The findings of Gui et al.^1^ provide experimental and functional evidence (via select tau residue phosphorylation) that GSK-3β is activated by *P. gingivalis*-LPS, potentially indirectly, because of inflammation generated via several pathways described above. GSK-3β, phosphorylation of specific tau protein residues, is a key topic in neurodegenerative diseases like AD because phosphorylation at certain residues can influence the function of tau protein and its role in disease progression.

In conclusion, the combined application of *P. gingivalis*-LPS and Aβ-conditioned medium from microglia (AL-MCM) to the neuronal cell line N2a was found to decrease synaptic molecules, at both the gene expression and protein levels within neuronal synapses. Dendrites, as protoplasmic extensions of the neuronal cell body, are responsible for receiving and processing electrochemical signals,^14^ from synapsing neurons. Given their vital role in memory,^14^ Gui et al.^1^ study suggests synapses are particularly vulnerable to excessive inflammation and the main perpetrators driving the inflammatory response in this investigation were *P. gingivalis*-LPS and Aβ.

The findings of Gui et al.^1^ emphasize the importance of infection, specifically from the aspects of periodontal disease, in inflammation and its role in synaptic dysfunction ultimately leading to declining memory. Moreover, their innovative use of conditioned medium collected from microglia^1^ and its subsequent application to N2a cells to investigate its effect on neurons provides an alternative framework for understanding the broader implications of chronic systemic infections on cognitive decline for future research.

Given the global burden of dementia, well-founded concerns remain about the relationship between frequent infections and Aβ_42_, which leads to excessive inflammation and memory loss. This ‘dangerous liaison’ creates a vicious cycle, driving the relentless progression of cognitive deficit in AD.
